# Supplementary material for: Synergetic regulation of SEI mechanics and crystallographic orientation for stable lithium metal pouch cells
Source: Nat Commun. 2024 May 25;15:4454. doi: 10.1038/s41467-024-48889-8 (PMC11126705; doi:10.1038/s41467-024-48889-8)
Supplement: Supplementary file 1 — Supplementary Information [file 41467_2024_48889_MOESM1_ESM.pdf]

Supplementary Information

Synergetic Regulation of SEI Mechanics and Crystallographic Orientation for Stable Lithium Metal Pouch Cells

Yanhua Zhang,[1] Rui Qiao,[1] Qiaona Nie,[1] Peiyu Zhao,[1] Yong Li,[2] Yunfei Hong,[1] Shengjie Chen,[1] Chao Li,[1] Baoyu Sun,[1] Hao Fan,[1] Junkai Deng,[1] Jingying Xie,[2] Feng Liu,[1] Jiangxuan Song[1,*]

[1]State Key Laboratory for Mechanical Behavior of Materials, Shaanxi International Research Center for Soft Matter, Xi'an Jiaotong University, Xi'an 710049, China

[2]State Key Laboratory of Space Power-Sources Technology, Shanghai Institute of Space Power-Sources, Shanghai 200000, China

*Corresponding author. Email: songjx@xjtu.edu.cn

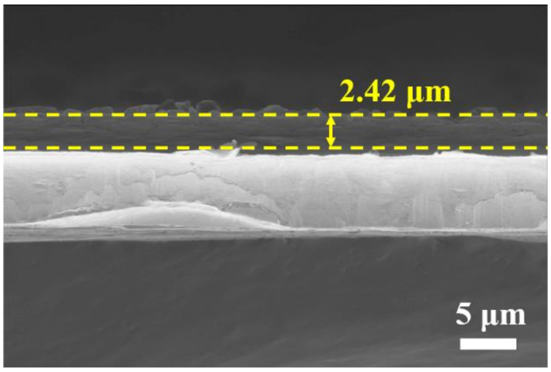

Supplementary Fig. 1. Cross-sectional SEM image of the YP-Cu electrode.

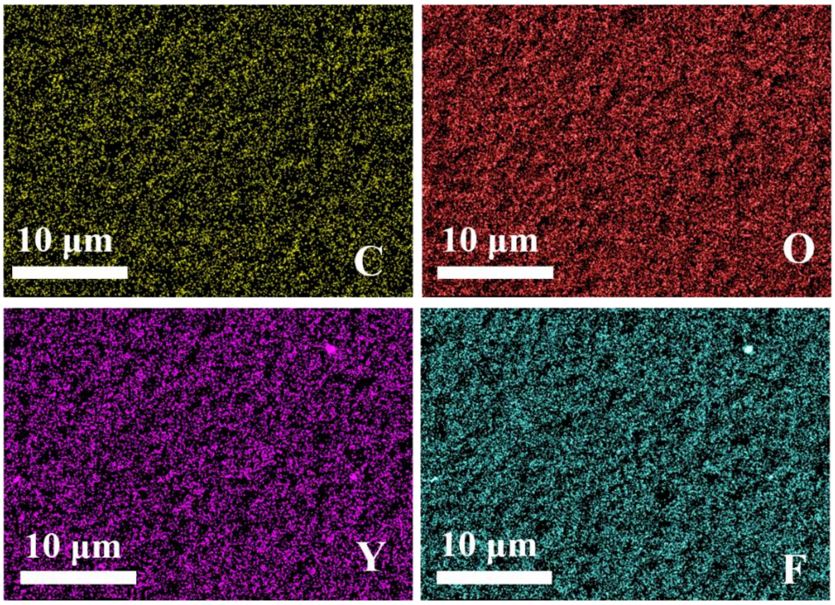

Supplementary Fig. 2. The EDS element mappings of C, O, Y, and F in the YP-Cu electrode.

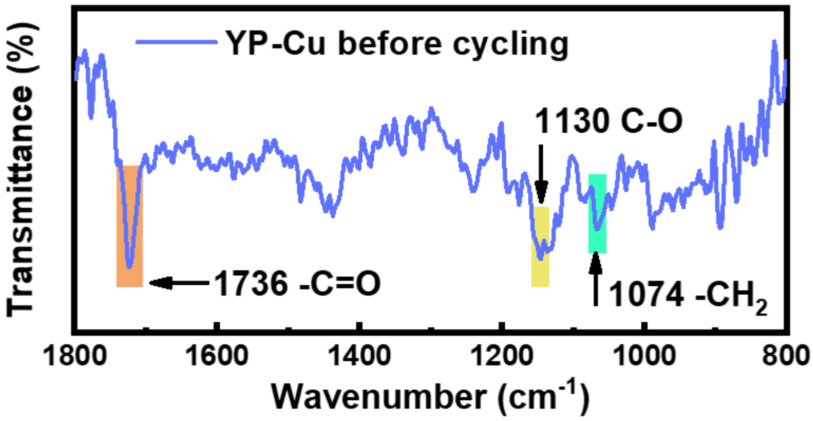

Supplementary Fig. 3. FTIR spectra of the YP-Cu before cycling.

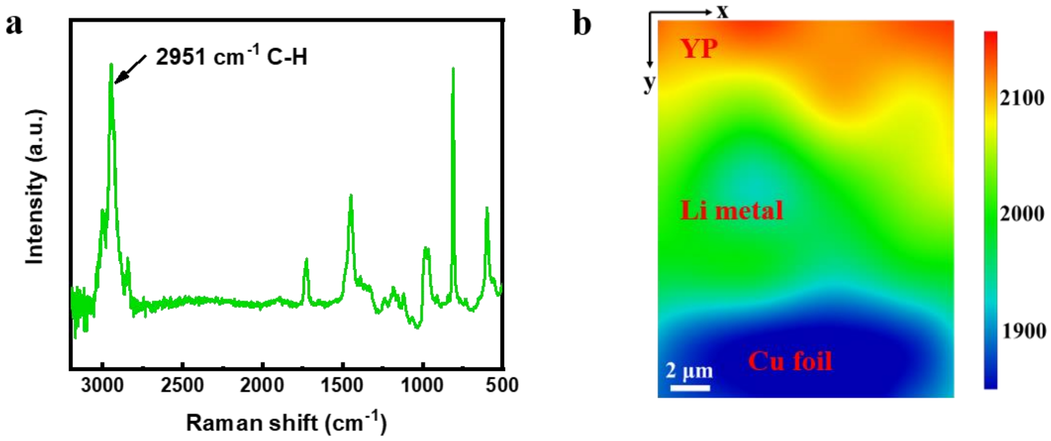

Supplementary Fig. 4. Raman spectra of (a) the PMMA and (b) 2D Raman spectra of YP-Cu anode tested at the Raman band of 2951 cm^{-1} after lithium plating of 2 mAh cm^{-2} at 1 mA cm^{-2}.

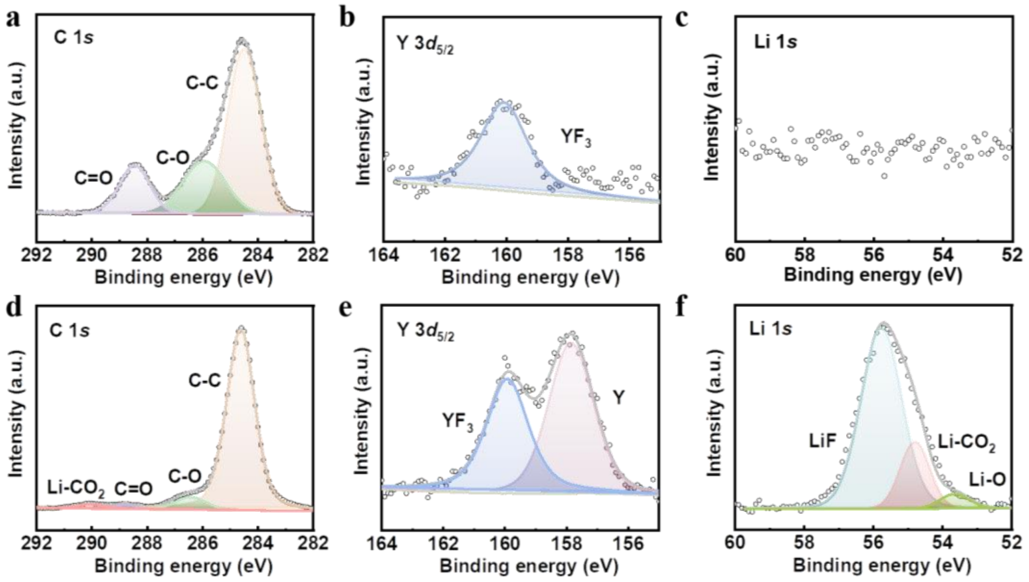

Supplementary Fig. 5. XPS spectra of C 1*s*, Y 3$d_{5/2}$, and Li 1*s* for the YP-Cu electrode (a-c) before and (d-f) after Li plating.

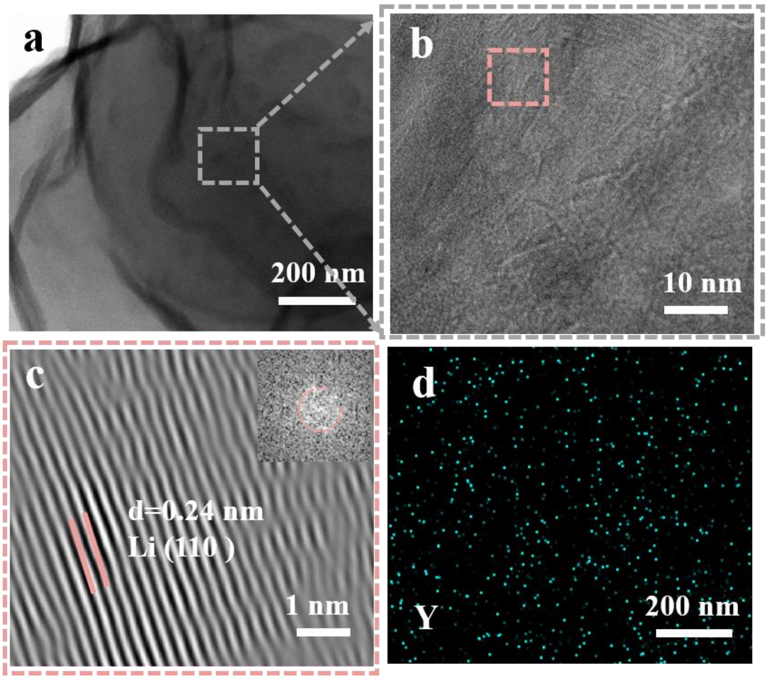

Supplementary Fig. 6. Cryo-TEM images of YP-Cu electrodes at (a) low-resolution and (b) enlarged-resolution, (c) corresponding HR-TEM and FFT images (inset), and (d) EDS elemental mappings of Y element.

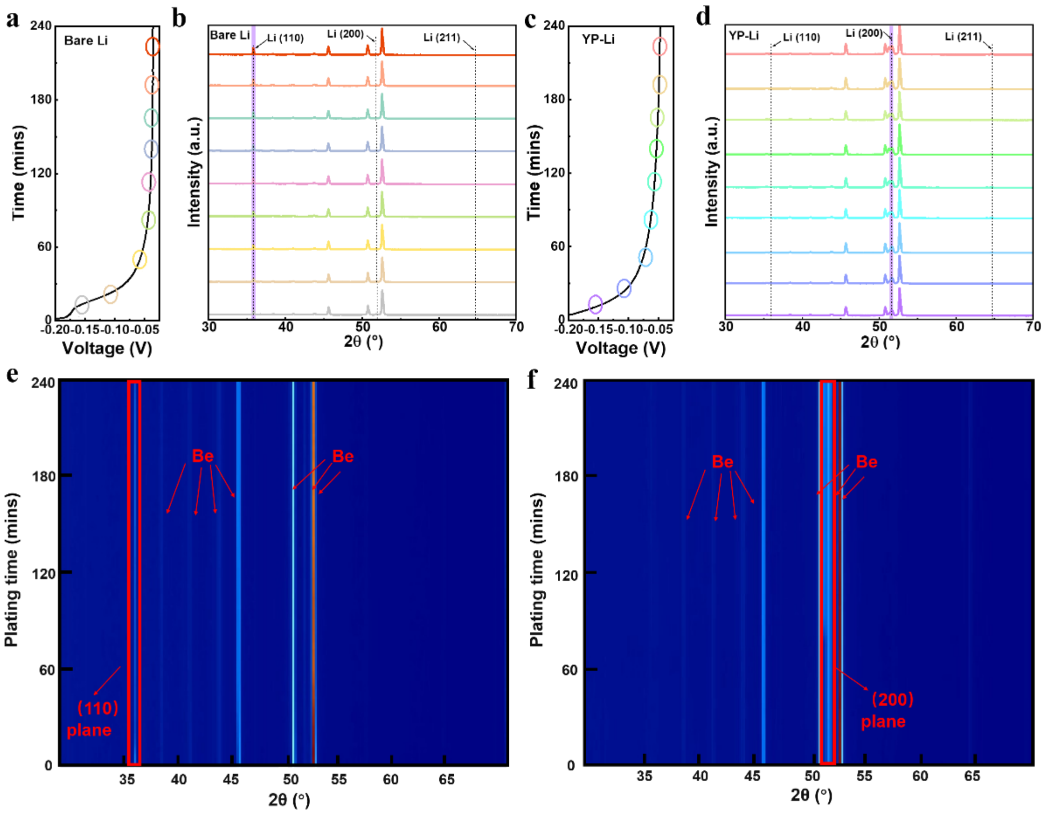

Supplementary Fig. 7. Typical discharge curves and *in situ* XRD patterns of (a, c) bare Li and (b, d) YP-Li. *In situ* synchrotron XRD contour map during Li deposition of (e) bare Li and (f) YP-Li.

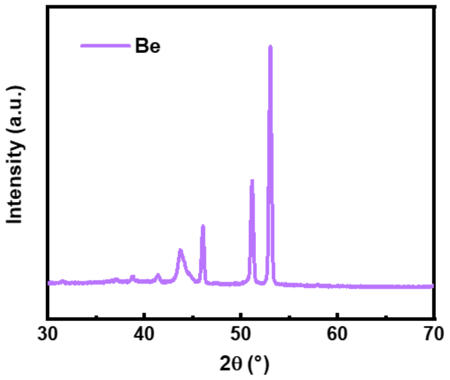

Supplementary Fig. 8. XRD pattern of Be.

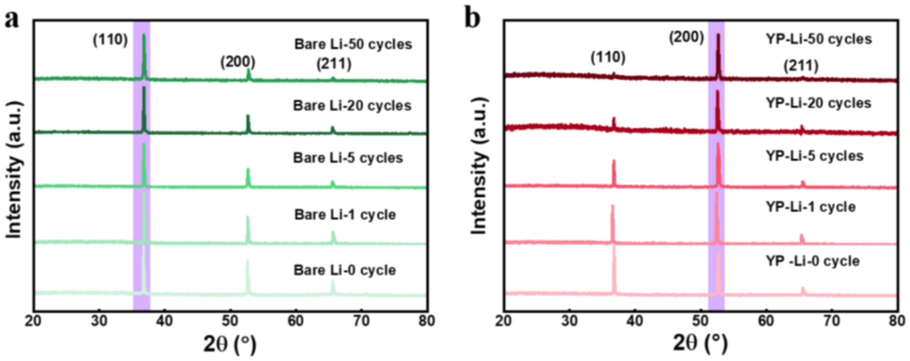

Supplementary Fig. 9. XRD patterns of the (a) bare Li and (b) YP-Li after different cycles.

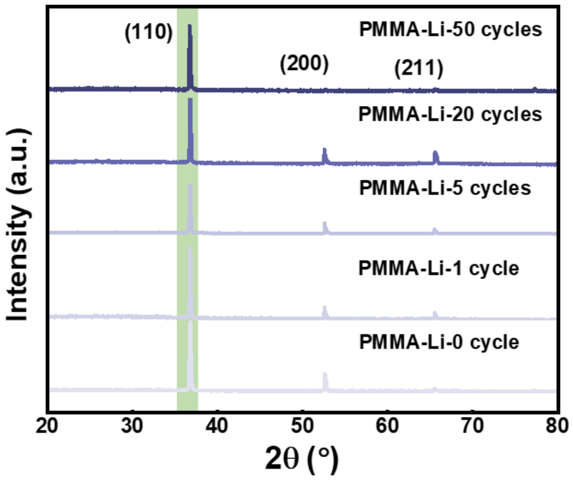

Supplementary Fig. 10. XRD patterns of the PMMA-Li after different cycles.

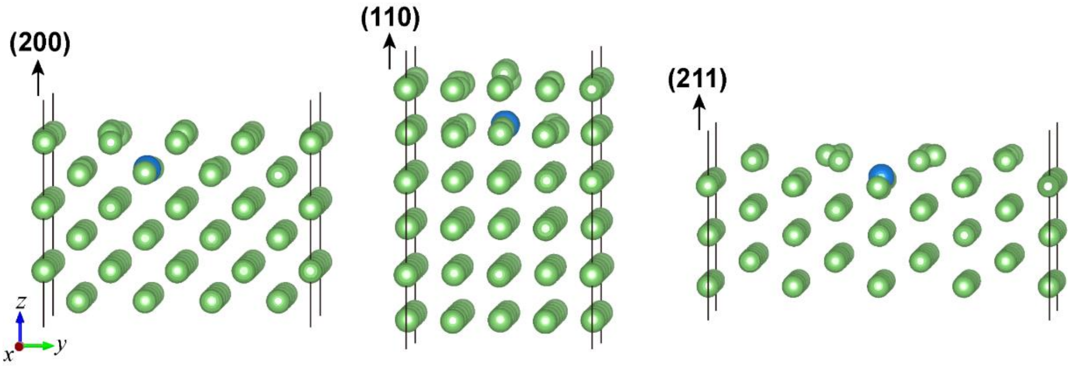

Supplementary Fig. 11. Model diagram with all atoms when Y doped to the second layer of Li metal.

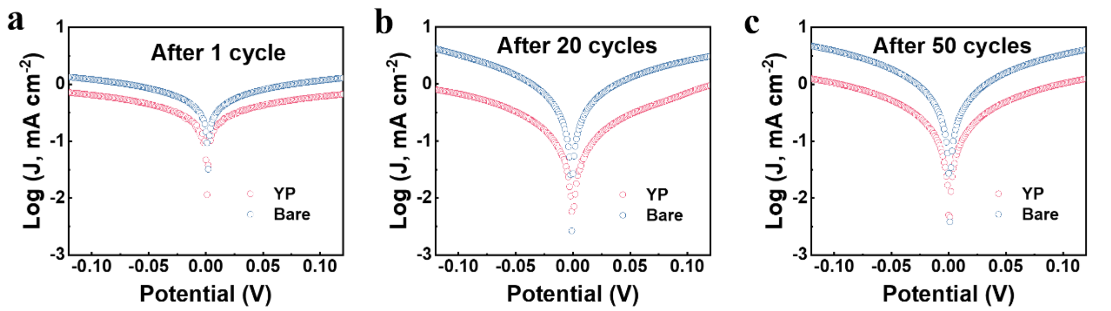

Supplementary Fig. 12. Potentiodynamic polarization curves with YP-Li and bare Li electrodes after (a) 1, (b) 20, and (c) 50 cycles.

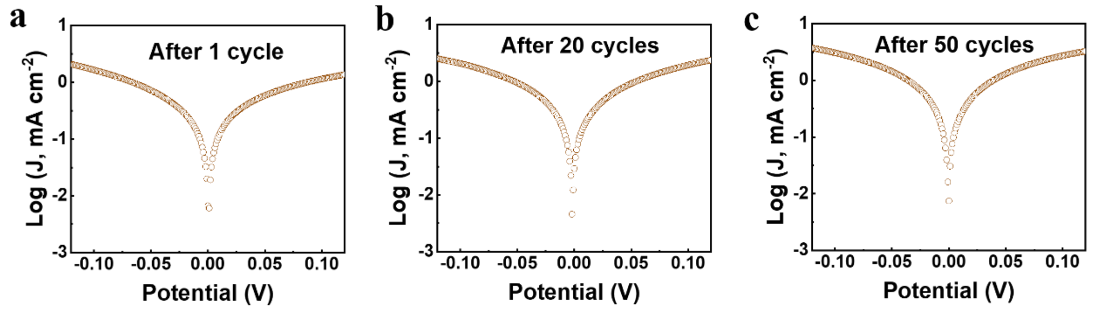

Supplementary Fig. 13. Potentiodynamic polarization curves with PMMA-Li electrodes after (a) 1, (b) 20, and (c) 50 cycles.

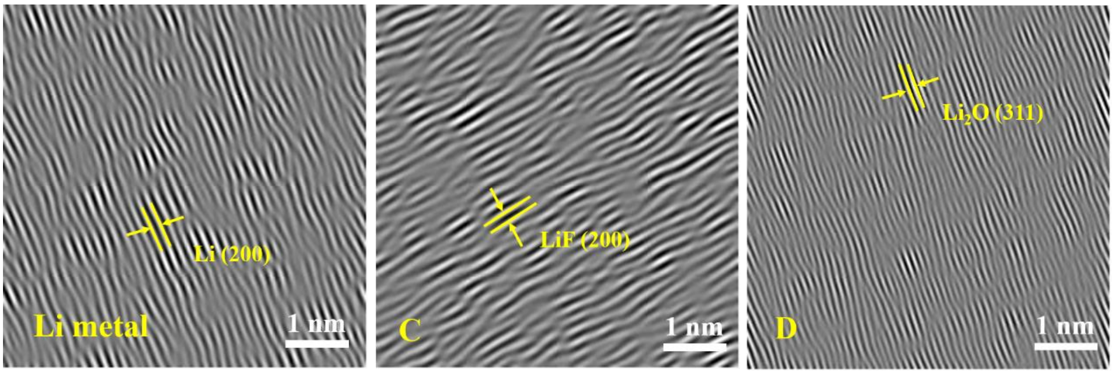

Supplementary Fig. 14. The corresponding local fast Fourier transform images of Fig. 4a.

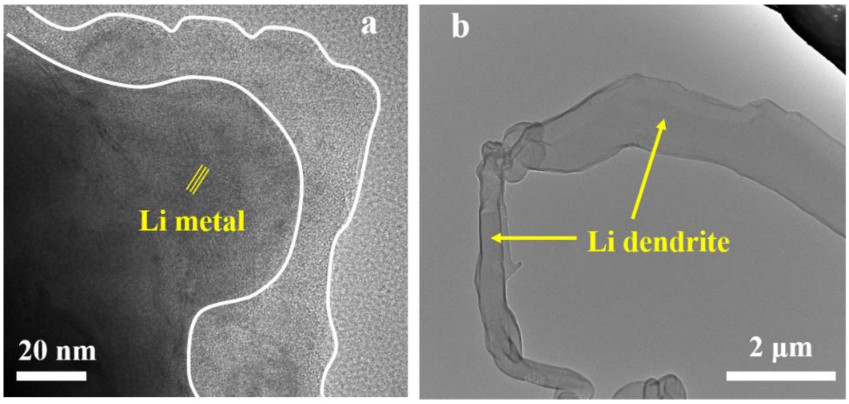

Supplementary Fig. 15. Cryo-TEM image of the bare Cu electrode at (a) high and (b) low resolution.

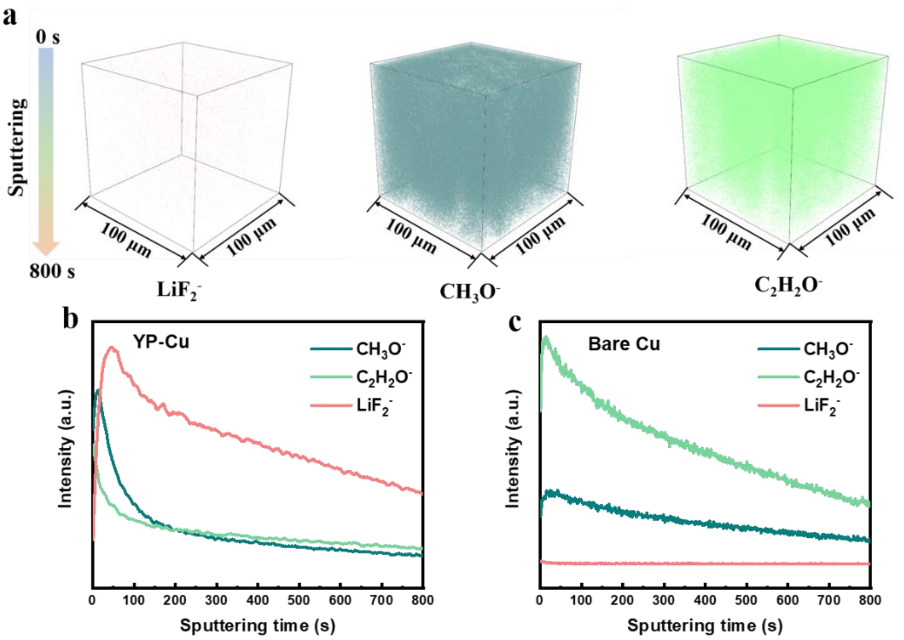

Supplementary Fig. 16. The 3D views of LiF_2^- (for LiF), CH_3O^-, and $C_2H_2O^-$ (for organic components) in the ToF-SIMS sputtered volumes of the (a) bare Cu induced SEI. The corresponding ToF-SIMS depth profiles for CH_3O^-, $C_2H_2O^-$, LiF_2^- in the (b) YP-Cu and (c) bare Cu induced SEI.

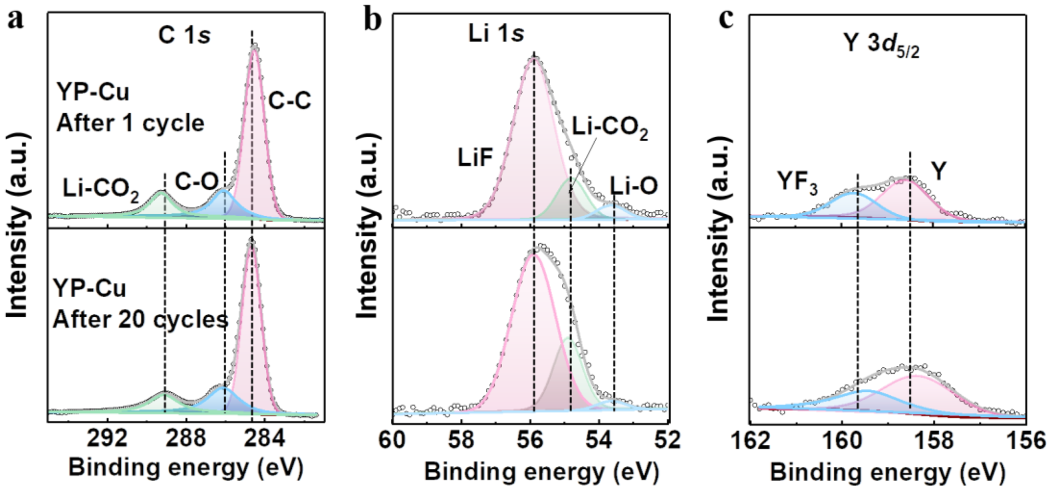

Supplementary Fig. 17. XPS spectra of (a) C 1*s*, (b) Li 1*s*, and (c) Y 3$d_{5/2}$ for YP-Cu after 1 cycle and 20 cycles with 4 mAh cm^{-2} at 1 mA cm^{-2}.

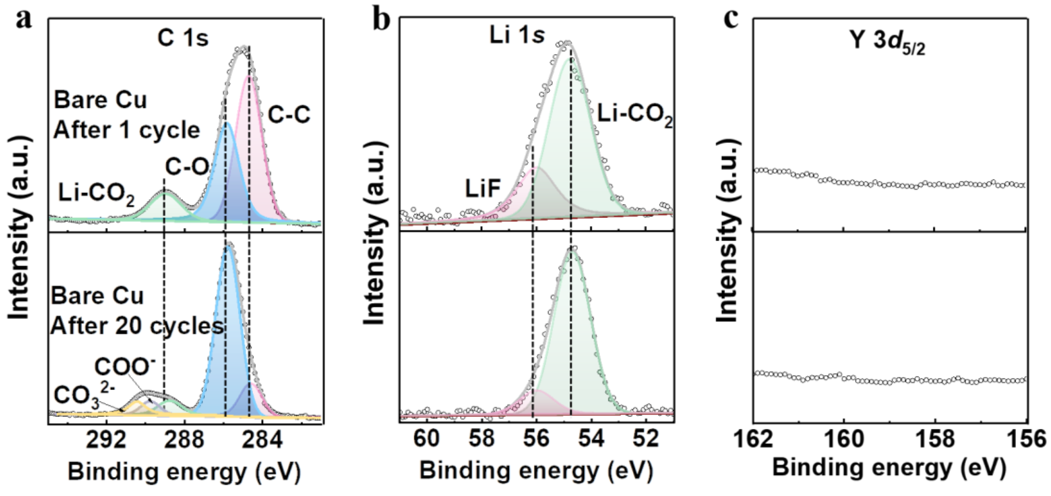

Supplementary Fig. 18. XPS spectra of (a) C 1*s*, (b) Li 1*s*, and (c) Y 3$d_{5/2}$ for bare Cu after 1 cycle and 20 cycles with 4 mAh cm^{-2} at 1 mA cm^{-2}.

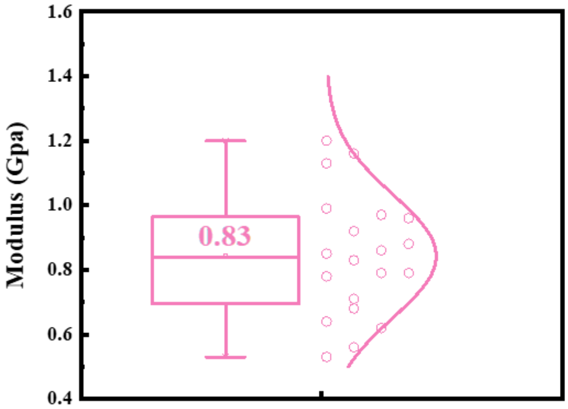

Supplementary Fig. 19. Young's modulus of YP-Cu electrode.

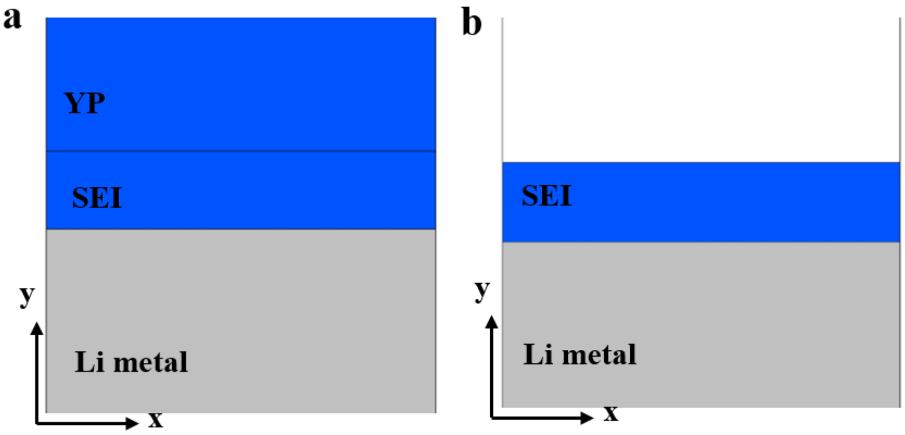

Supplementary Fig. 20. Stress distribution for (a) YP-Cu and (b) bare Cu electrodes at early stage (0 s).

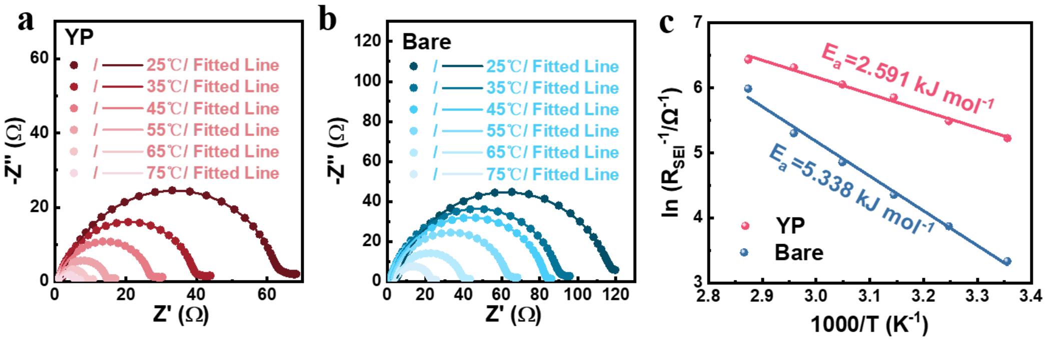

Supplementary Fig. 21. Nyquist plots at various temperature (a) with and (b) without YP. (c) Arrhenius plots with and without YP and the calculated activation energy for Li^+ diffusion through SEI.

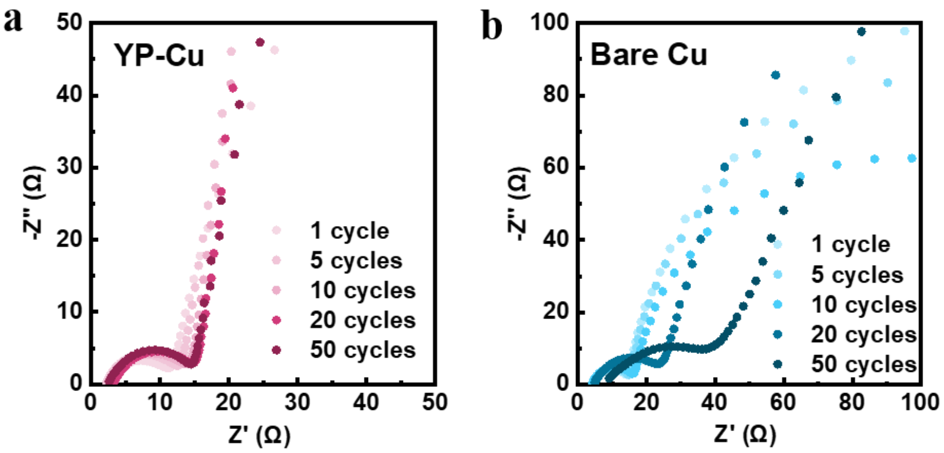

Supplementary Fig. 22. Nyquist plots for (a) YP-Cu and (b) bare Cu tested at different cycles.

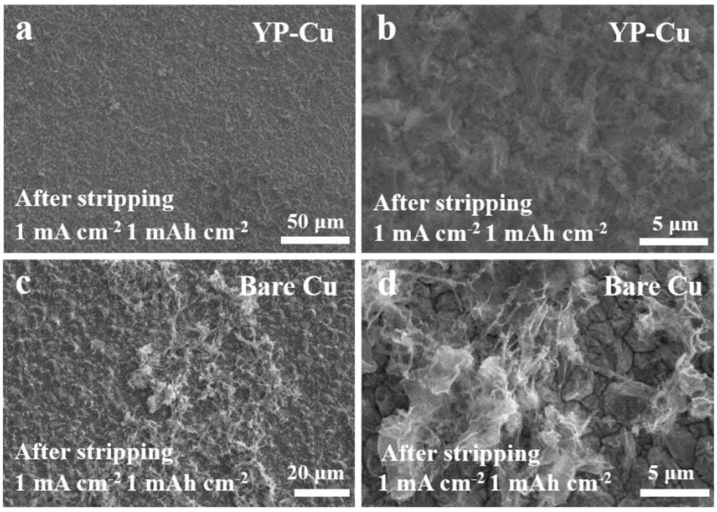

Supplementary Fig. 23. Top-view morphology of Li stripping from (a-b) YP-Cu and (c-d) bare Cu electrodes at 1 mA cm^{-2} for 1 mAh cm^{-2}.

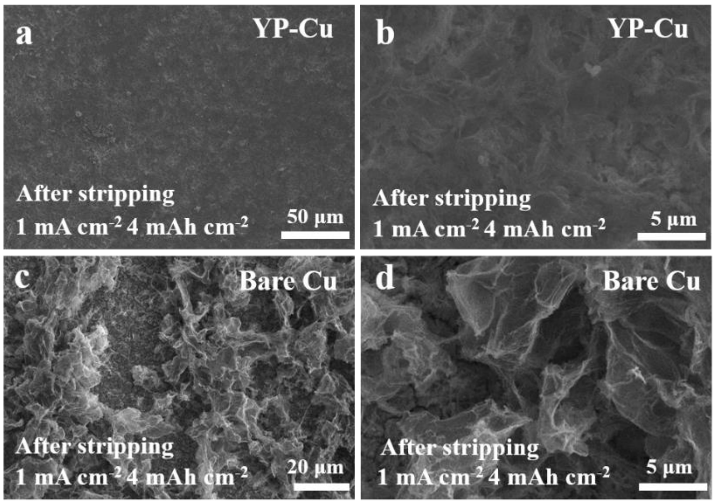

Supplementary Fig. 24. Top-view morphology of Li stripping from (a-b) YP-Cu and (c-d) bare Cu electrodes at 1 mA cm^{-2} for 4 mAh cm^{-2}.

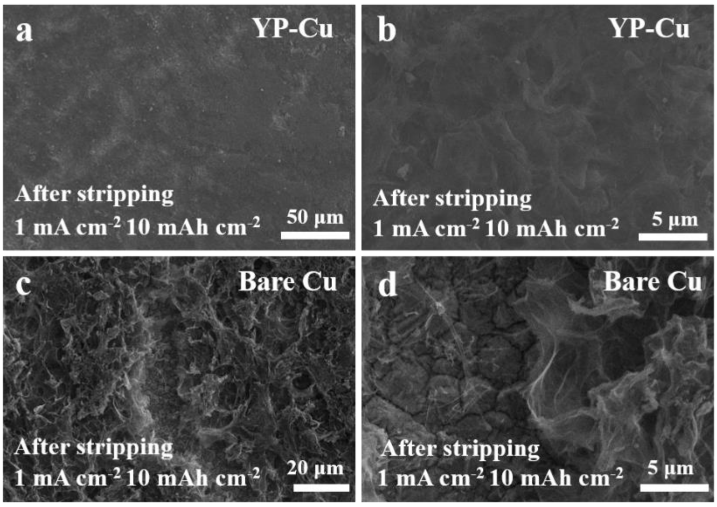

Supplementary Fig. 25. Top-view morphology of Li stripping from (a-b) YP-Cu and (c-d) bare Cu electrodes at 1 mA cm^{-2} for 10 mAh cm^{-2}.

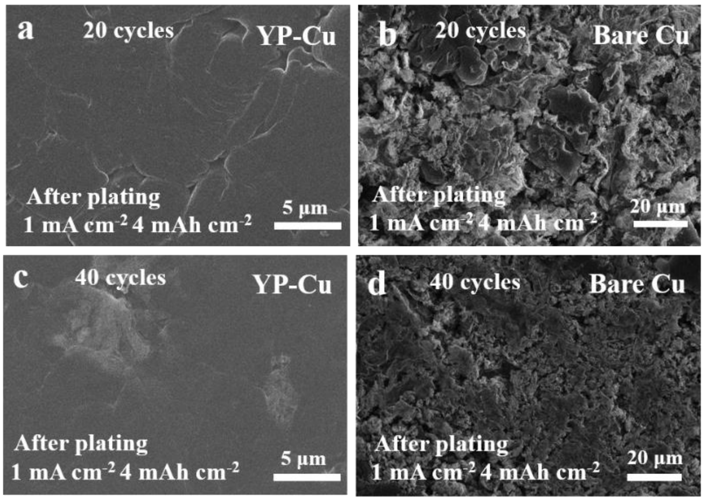

Supplementary Fig. 26. Top-view SEM images of (a, c) YP-Cu and (b, d) bare Cu at the plating process after (a-b) 20 and (c-d) 40 cycles with areal capacity of 4 mAh cm^{-2} at 1 mA cm^{-2}.

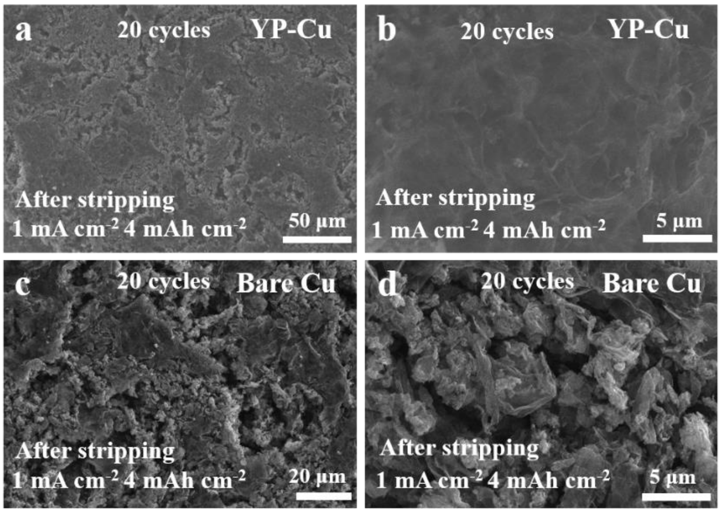

Supplementary Fig. 27. Top-view morphology of Li stripping from (a-b) YP-Cu and (c-d) bare Cu electrodes after 20 cycles at 1 mA cm^{-2} for 4 mAh cm^{-2}.

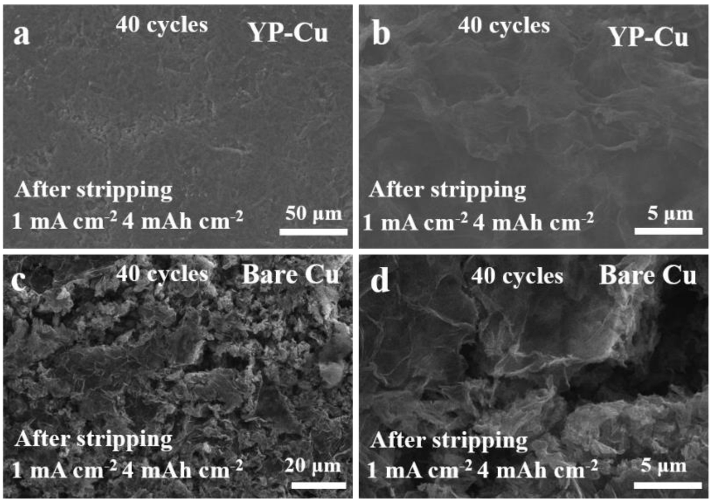

Supplementary Fig. 28. Top-view morphology of Li stripping from (a-b) YP-Cu and (c-d) bare Cu electrodes after 40 cycles at 1 mA cm^{-2} for 4 mAh cm^{-2}.

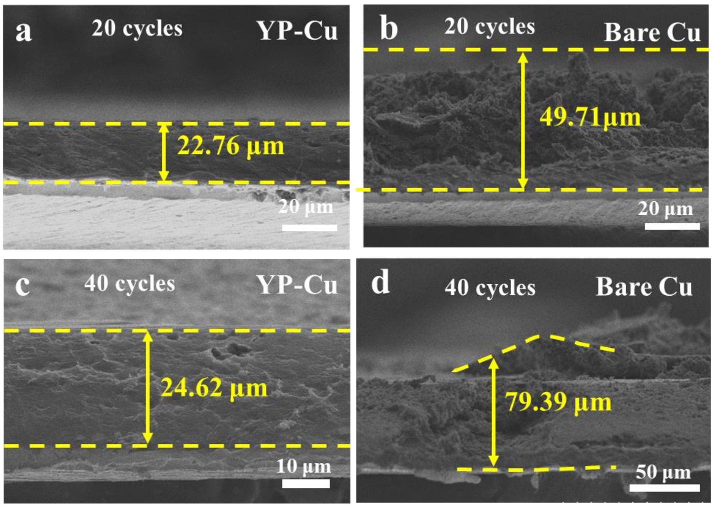

Supplementary Fig. 29. Cross-sectional SEM images of YP-Cu and bare Cu at the plating process after (a-b) 20 and (c-d) 40 cycles with areal capacity of 4 mAh cm^{-2} at 1 mA cm^{-2}.

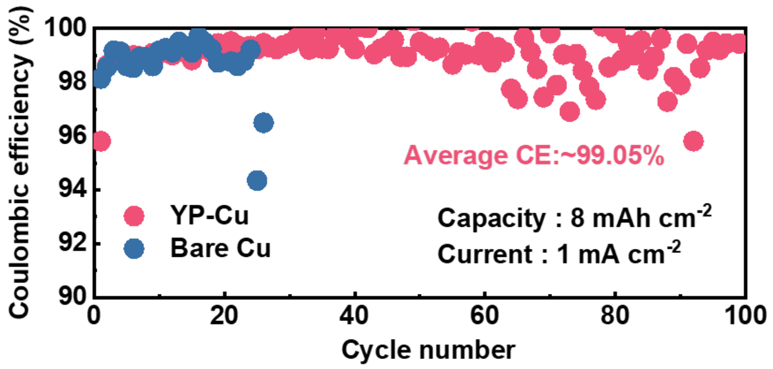

Supplementary Fig. 30. Cycling performance of Li||YP-Cu and Li||Cu asymmetric cells with 8 mAh cm^{-2} at 1 mA cm^{-2}.

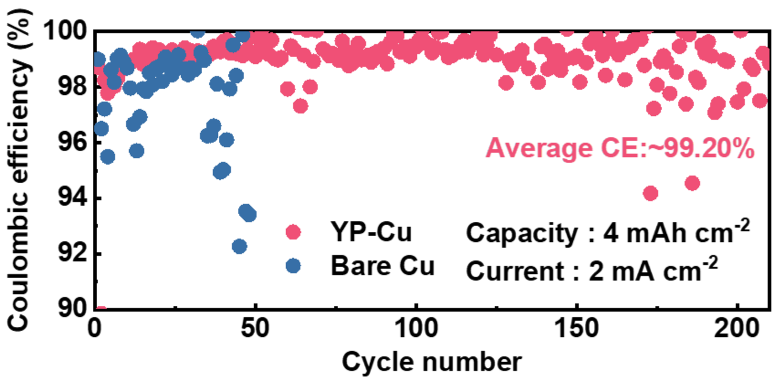

Supplementary Fig. 31. Cycling performance of Li||YP-Cu and Li||Cu asymmetric cells with 4 mAh cm^{-2} at 2 mA cm^{-2}.

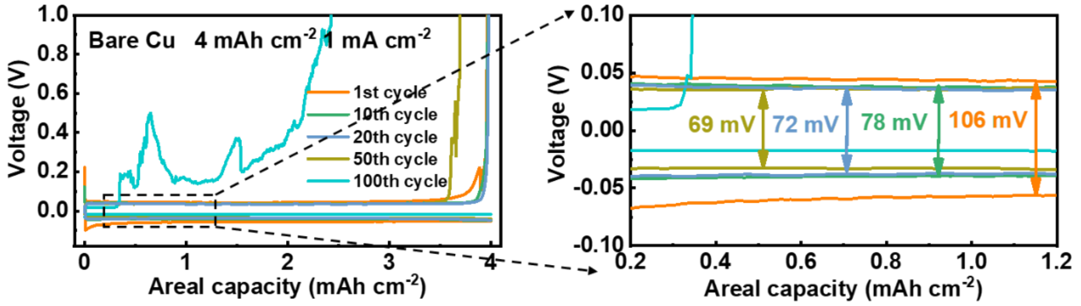

Supplementary Fig. 32. Voltage profiles of Li||Cu asymmetric cell with 4 mAh cm^{-2} at 1 mA cm^{-2}.

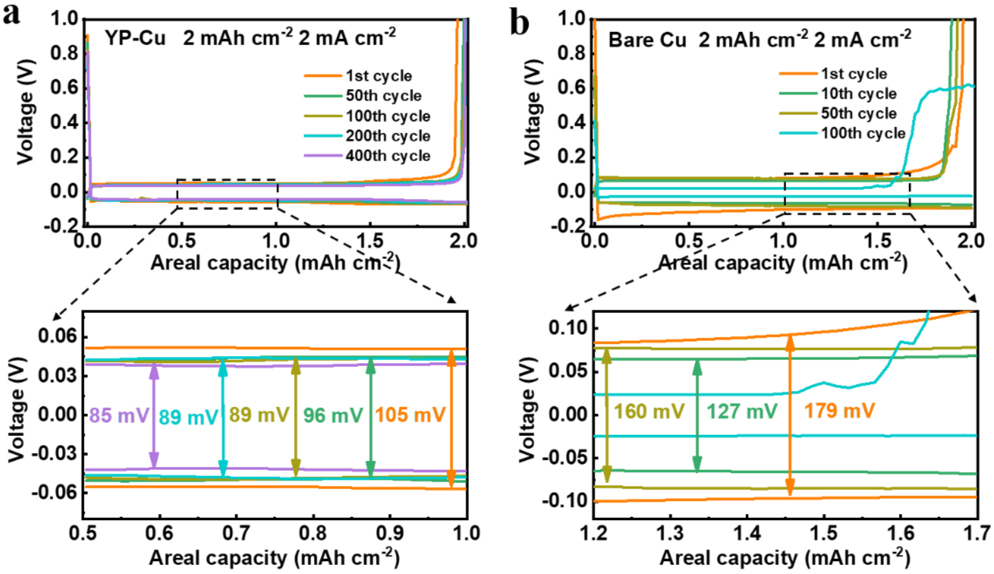

Supplementary Fig. 33. Voltage profiles of (a) Li||YP-Cu and (b) Li||Cu asymmetric cells with 2 mAh cm^{-2} at 2 mA cm^{-2}.

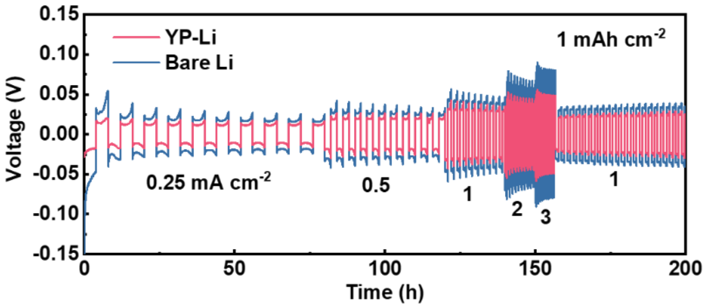

Supplementary Fig. 34. Voltage profiles of YP-Li||YP-Li and Li||Li symmetrical cells at various current densities with a fixed capacity of 1 mAh cm^{-2}.

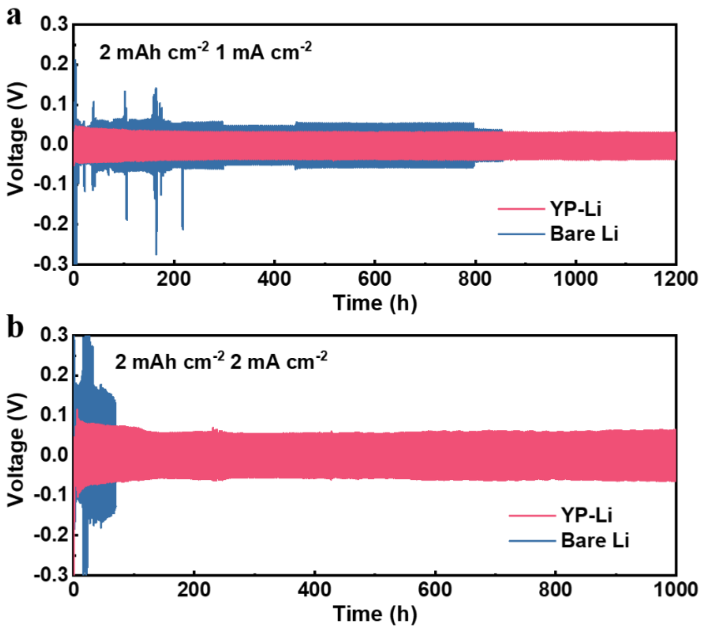

Supplementary Fig. 35. Cycling stability of YP-Li||YP-Li and Li||Li symmetrical cells at different current density of (a) 1 mA cm^{-2} and (b) 2 mA cm^{-2} with an areal capacity of 2 mAh cm^{-2}.

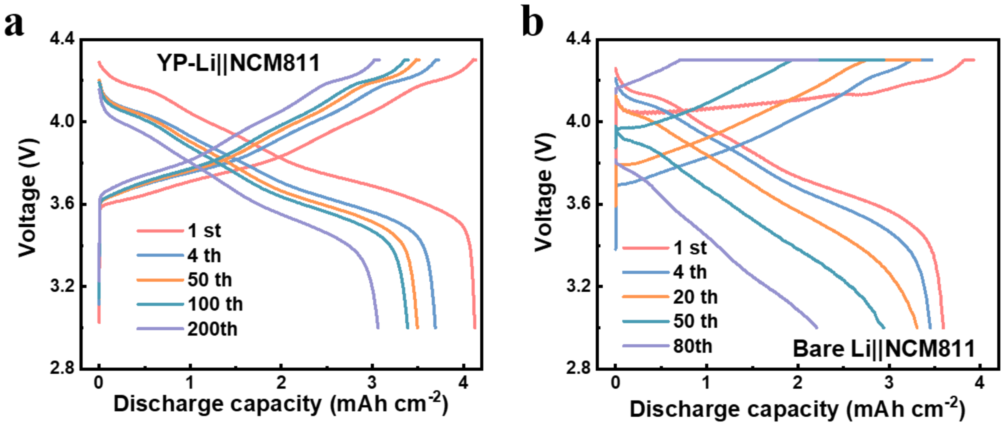

Supplementary Fig. 36. The charge-discharge voltage profiles of (a) YP-Li||NCM811 and (b) bare Li||NCM811 full cell at 1 C.

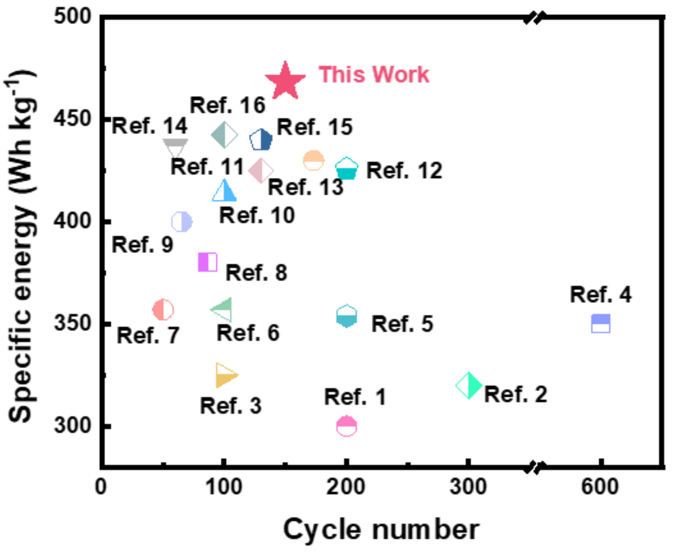

Supplementary Fig. 37. The comparison performance of of high-energy-density (over 300 Wh kg^{-1}) Ah-type Li metal pouch cells in published literatures and this work.

Supplementary Table 1. Total energy for the specific surface in the slab structures.

	Atom	E_{total} $(eV/atom)$	S (Å^2)	$\bar{E}_S$ $(meV/Å^2)$
**Li (110)**	96	-1.8171	136.1426	31.30
**Li (211)**	96	-1.7424	227.7913	34.17
**Li (200)**	96	-1.7856	195.3471	39.17
**$Li_{95}Y$ (110)1st**	96	-1.8511	136.1426	33.48
**$Li_{95}Y$ (211)1st**	96	-1.7740	227.7913	36.57
**$Li_{95}Y$ (200)1st**	96	-1.8205	195.3471	20.35
**$Li_{95}Y$ (110)2nd**	96	-1.8574	136.1426	28.99
**$Li_{95}Y$ (211)2nd**	96	-1.7792	227.7913	34.40
**$Li_{95}Y$ (200)2nd**	96	-1.8230	195.3471	19.14

Supplementary Table 2. The detailed parameters of a high-energy YP-Li||NCM811 pouch cell.

Cell component	Cell parameters	Value
Cathode	Materials	NCM811
	Number	7
	Active material loading (%)	0.955
	Area weigh (each side, mg/cm^2)	30.0
	Area capacity (each side, mAh/cm^2)	6
	Electrode length (mm)	56
	Electrode width (mm)	80
	NCM811 Weight (g)	18.82
	The Weight of Al foil (g)	1.05
	The Weight of Al foil + NCM811 (g)	19.87
Anode	Materials	YP-Li
	Number	8
	Li thickness (double sides, μm)	100
	Area capacity (each side, mAh/cm^2)	10
	N/P ratio	1.67
	Weight (g)	2.32
Electrolyte	E/C ratio (g/Ah)	1.98
	Weight (g)	8.32
Separator	Weight (g)	1.36
Package and lugs	Weight (g)	2.20
Pouch cell	Pressure (kPa)	250
	Mid-value voltage (V)	3.8
	Capacity (Ah)	4.2
	Total Weigh (g)	34.07
	Energy density (Wh/kg)	$\frac{Capacity\ (Ah)\ \times\ Voltage\ (V)}{Total\ Weight\ (kg)} = \frac{4.2\ Ah \times 3.8\ V}{0.03407\ (kg)} = 468$

Supplementary Table 3. Material properties and parameters.

Physical parameters	Symbol	Value
Initial electrolyte concentration	C_0	1000 mol m^{-3}
Exchange current density	i_0	100 A m^{-2}
Electrolyte diffusion coefficient[17]	D_e	1×10^{-9} m^2 s^{-1}
SEI diffusion coefficient[17]	D_{SEI}	1×10^{-11} m^2 s^{-1}
PMMA diffusion coefficient	D_{PMMA}	1×10^{-11} m^2 s^{-1}
Young's modulus of SEI[17]	E_{SEI}	3 GPa
Young's modulus of PMMA	E_{PMMA}	0.8 GPa
Conductivity of Li	σ_{Li}	1.07×10^7 S m^{-1}
Conductivity of Y	σ_Y	1.66×10^6 S m^{-1}

References

1. Niu, C., et al. High-energy lithium metal pouch cells with limited anode swelling and long stable cycles. *Nat. Energy* **4**, 551-559 (2019).
2. Qiao, Y., et al. A high-energy-density and long-life initial-anode-free lithium battery enabled by a Li_2O sacrificial agent. *Nat. Energy* **6**, 653-662 (2021).
3. Gao, Y., et al. Effect of the supergravity on the formation and cycle life of non-aqueous lithium metal batteries. *Nat. Commun.* **13**, 5 (2022).
4. Niu, C., et al. Balancing interfacial reactions to achieve long cycle life in high-energy lithium metal batteries. *Nat. Energy* **6**, 723-732 (2021).
5. Chang, Z., et al. An improved 9 micron thick separator for a 350 Wh/kg lithium metal rechargeable pouch cell. *Nat. Commun.* **13**, 6788 (2022).
6. Kwon, H., et al. Weakly coordinated Li ion in single-ion-conductor-based composite enabling low electrolyte content Li-metal batteries. *Nat. Commun.* **14**, 4047 (2023).
7. Huang, K., et al. Regulation of SEI formation by anion receptors to achieve ultra-stable lithium-metal batteries. *Angew. Chem. Int. Ed.* **60**, 19232-19240 (2021).
8. Tan, Y.-H., et al. Lithium fluoride in electrolyte for stable and safe lithium-metal batteries. *Adv. Mater.* **33**, 2102134 (2021).
9. Zhang, K., et al. A high-performance lithium metal battery with ion-selective nanofluidic transport in a conjugated microporous polymer protective layer. *Adv. Mater.* **33**, e2006323 (2021).

10. Zhang, M., et al. Boosting the temperature adaptability of lithium metal batteries via a moisture/acid-purified, ion-diffusion accelerated separator. *Adv. Energy Mater.* **12**, 2201390 (2022).
11. Zhang, Y., et al. Enabling 420 Wh kg^{-1} stable lithium metal pouch cells by lanthanum doping. *Adv. Mater.* **35**, 2211032 (2023).
12. Zhang, G., et al. A monofluoride ether-based electrolyte solution for fast-charging and low-temperature non-aqueous lithium metal batteries. *Nat. Commun.* **14**, 1081 (2023).
13. Zhang, Q.-K., et al. Reforming the uniformity of solid electrolyte interphase by nanoscale structure regulation for stable lithium metal batteries. *Angew Chem. Int. Ed.* **62**, e202306889 (2023).
14. Zhang, S., et al. In situ-polymerized lithium salt as a polymer electrolyte for high-safety lithium metal batteries. *Energy Environ. Sci.* **16**, 2591-2602 (2023).
15. Zhang, Q.-K., et al. Homogeneous and mechanically stable solid–electrolyte interphase enabled by trioxane-modulated electrolytes for lithium metal batteries. *Nat. Energy* **8**, 725-735 (2023).
16. Mao, M., et al. Anion-enrichment interface enables high-voltage anode-free lithium metal batteries. *Nat. Commun.* **14**, 1082 (2023).
17. Shen, X., et al. The Failure of Solid Electrolyte Interphase on Li Metal Anode: Structural Uniformity or Mechanical Strength? *Adv. Energy Mater.* **10**, 1903645 (2020).
